# In Search of an Uncultured Human-Associated TM7 Bacterium in the Environment

**DOI:** 10.1371/journal.pone.0021280

**Published:** 2011-06-20

**Authors:** Jorge M. Dinis, David E. Barton, Jamsheed Ghadiri, Deepa Surendar, Kavitha Reddy, Fernando Velasquez, Carol L. Chaffee, Mei-Chong Wendy Lee, Helen Gavrilova, Hazel Ozuna, Samuel A. Smits, Cleber C. Ouverney

**Affiliations:** 1 Department of Biological Sciences, San Jose State University, San Jose, California, United States of America; 2 Department of Zoology, University of Florida, Gainesville, Florida, United States of America; 3 Department of Biomolecular Engineering, University of California Santa Cruz, Santa Cruz, California, United States of America; 4 Science and Technology Department, Universidad Metropolitana, San Juan, Puerto Rico; University of Wisconsin-Milwaukee, United States of America

## Abstract

We have identified an environmental bacterium in the Candidate Division TM7 with ≥98.5% 16S rDNA gene homology to a group of TM7 bacteria associated with the human oral cavity and skin. The environmental TM7 bacterium (referred to as TM7a-like) was readily detectable in wastewater with molecular techniques over two years of sampling. We present the first images of TM7a-like cells through FISH technique and the first images of any TM7 as viable cells through the STARFISH technique. *In situ* quantification showed TM7 concentration in wastewater up to five times greater than in human oral sites. We speculate that upon further characterization of the physiology and genetics of the TM7a-like bacterium from environmental sources and confirmation of its genomic identity to human-associated counterparts it will serve as model organisms to better understand its role in human health. The approach proposed circumvents difficulties imposed by sampling humans, provides an alternative strategy to characterizing some diseases of unknown etiology, and renders a much needed understanding of the ecophysiological role hundreds of unique Bacteria and Archaea strains play in mixed microbial communities.

## Introduction

The Bacteria Domain experienced an explosion of novel lineages identified within the last decade, especially of lineages made entirely of uncultured members [1]. Since molecular approaches were applied to identify uncultured microbes, the number of Bacteria phyla increased from 12 (none uncultured) in 1987 [2] to about 52 in 2003 [3], half of which were termed *candidate division* since all of its members were yet uncultured. The proportion of candidate divisions is expected to increase as more prokaryotic DNA sequences are generated and today the NCBI Taxonomy Database (not peer-reviewed) lists 24 Bacteria phyla (those with at least some cultured species), 70 candidate divisions, and many unclassified entries. For this paper, we will capitalize Bacteria to refer to the domain and use bacteria to refer to members within the domain Bacteria.

Since numerous cultivable bacteria have been shown to be instrumental in human development [4], health [5] and diseases [6], [7], it is reasonable to speculate that strains from uncultured groups, which comprise nearly 80% of the human gut [8] and 68% of human oral [9] microbial consortia, participate in similar functions. The study of human-associated uncultured prokaryotes, however, has many practical limitations, such as access to patient samples, unpredictable microbial composition, and low relative abundance, all of which challenge experimental promptness and reproducibility.

The Bacteria Candidate Division TM7 was first reported in 2001 in diverse environmental sources including soil, freshwater, seawater, hot springs, mouse feces, and termite guts [10]. Recently, TM7 has been detected in various human body sites [9], [11], [12], [13] and associated with the diseases periodontitis [14], vaginosis [15], and inflammatory bowel disease [16]. Yet, nothing is known about the direct role TM7 bacteria play in human health.

Although the 16S rDNA gene does not perfectly predict genomic wide phylogenetic homology, nor does it account for possible genomic differences caused by horizontal gene transfer, it is the most reliable and widely applied gene to predict basic genomic similarities [17], [18]. Valuable insight into core cell functions such as metabolic processes [19] may therefore be derived. In this study, we assume 16S rDNA gene similarities between phylotypes correspond to similarities in genomic content.

## Results

### Clone Libraries

Analyses from seven independent 16S rDNA gene clone libraries generated from activated wastewater (sludge) samples collected between January 2007 and December 2009 identified 153 clones in the Candidate Division TM7, of which 103 (67.3%) shared species-level homology (≥98.5% identity) with a cluster of human-associated TM7 phylotypes and 50 TM7 representatives that lacked such homology. Each library used freshly collected samples and all contained the TM7 model 16S rDNA gene as confirmed through DNA sequencing.

Activated wastewater clone AAWS56C (accession number HM208134) showed 99.7% identity to the human skin clone HM269723 [11] and 98.6% identity to the human oral TM7 clone TM7a (AY144355) [14] based on the percent identity from our manually refined ARB multi-sequence alignment (MSA). Screening of lab members for potential sample contamination (see methods) detected TM7 in 4/10 oral and in 0/50 skin samples. The four TM7 positive oral samples generated 150 clones belonging to TM7 (based on MSA) of which 13 (8.7%) clones had species-level (≥98.5%) 16S rDNA identity to our sludge clone AAWS56C. Relevant and unique 16S rDNA sequences were deposited in GenBank with accession numbers for 6 sludge (HM208132-37) and 14 human oral (HM215440-53) clones. A nucleotide-base comparison among sludge and human homologous sequences (Figure S1, Supporting information) revealed conserved base mutations and various single-nucleotide polymorphic sites that differentiated the sludge sequences from the human-counterparts, dismissing potential sample contamination.

### Phylogenetic Analysis

**Figure 1:**
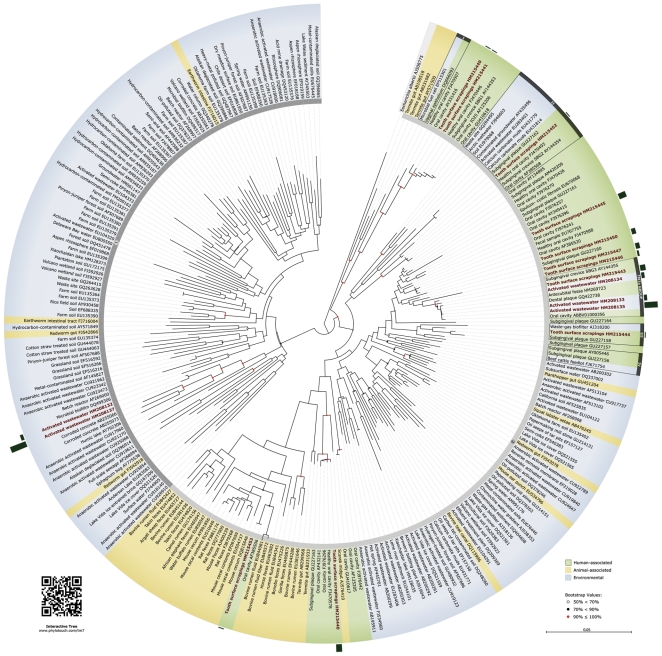
Neighbor-joining phylogenetic tree of Candidate Division TM7 with 255 TM7 phylotypes, where 160 are identified as being environmental, 42 as animal-associated, and 53 as human-associated, with a total of 208 unique OTUs. The bar chart above the outer ring represents the clone counts for each of the 16 phylotypes in our study. Subdivisions 1 and 2 are marked with a gray banner on the inner border of ring. Bootstrap values of major branches are indicated according to legend. An interactive version of this tree (http://www.phylotouch.com/tm7), developed with jsPhyloSVG [36], includes meta-analysis data such as distance matrices and links to sequences and publications.

We established the evolutionary relationships among 16 of our TM7 sequences (5 from sludge and 11 from oral samples) against 239 publicly available TM7 reference sequences from a wide-range of environmental, animal, and human sources (Figure 1). With support of bootstrap resampling, two monophyletic TM7 subdivisions emerged (Figure 1, regions labeled 1 and 2, inner rings). Subdivision 1 was characterized by phylotypes predominantly isolated from environmental sources (soil, rhizosphere, marine, and freshwater), whereas subdivision 2 included phylotypes from environmental, activated wastewater, animal (non-human), and human sources. Three of our activated sludge clones (HM208133, HM208134, and HM208135) grouped with human oral and skin phylotypes, including TM7a (Figure 1, Enclosure “TM7a Group”), the human oral-associated TM7 with the most complete genome [20]. Hence we will refer to our TM7 phylotypes closest to the human TM7a as “TM7a-like” or “environmental TM7a.”

UniFrac statistical analyses were used to determine the relationships of the TM7 phylotypes from the three sample sources or “communities” (UniFrac terminology) in Figure 1: (E) environmental sites, (A) animals, and (H) humans. The UniFrac ‘environmental distance matrix,’ which compares each sample site to the other and measures the evolutionary history shared between each pair of sample sites, showed that for all phylotypes included in Figure 1, the TM7 associated with animals (A) were more similar to the TM7 associated with humans (H) (UniFrac value between A and H = 0.8711) than either A or H was to the TM7 from E. UniFrac values between A and E = 0.9331 and between H and E = 0.9584. A smaller UniFrac value indicates communities that are more similar [21]. Testing for significant differences in evolutionary history within one community against any of the other two communities through the UniFrac ‘significant test for each individual community,’ showed that each community was highly significantly different from one another (P<0.001, P corrected for multiple comparisons).

Despite limited sampling, UniFrac ‘G-test for lineage-specific analysis,’ [21] to determine any community difference present within a particular lineage of the tree, showed that the eight phylotypes within the TM7a Group enclosure (five human-associated and three from environmental sites) were not significantly different from one another (P = 0.6840, P corrected for multiple comparisons). Similarly, no significant difference was found for lineage-specific analysis of eleven phylotypes within enclosures II (P = 0.8107), three phylotypes in enclosure III (P = 0.6260), and three phylotypes in enclosure IV (P = 0.62560) (Figure 1). Note that the Figure 1 focuses on TM7 sequences in our wastewater and in our human contamination control samples with highest similarity to previously discovered human-associated TM7. In addition, we included only representative TM7 phylotypes from lineages that did not share the human-environment homology.

### Quantitative PCR

**Figure 2:**
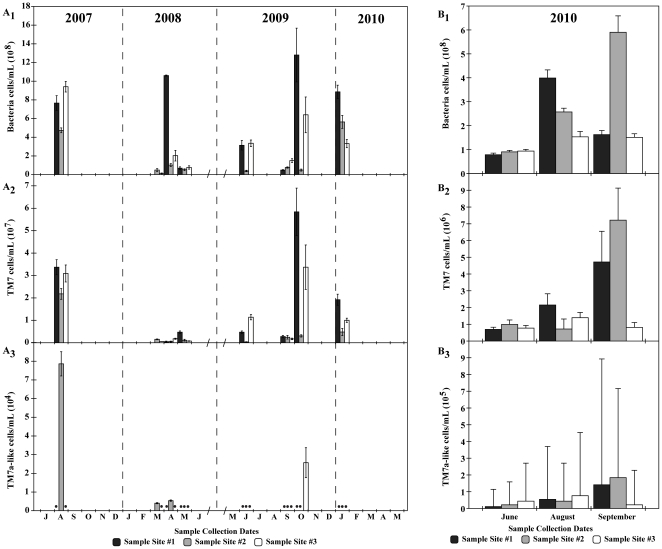
Quantification of Bacteria, TM7, and TM7a-like phylotypes by (A) qPCR and (B) FISH. Average number of (**A1** and **B1**) Bacteria cells mL^−1^, (**A2** and **B2**) Candidate Division TM7 cells mL^−1^, and (**A3** and **B3**) TM7a-like cells mL^−1^. Closed circles indicate undetermined quantification values. Error bars represent standard error of the mean.

Quantitative PCR (qPCR) assay, also targeting 16S rDNA, provided independent evidence supporting the prevalence of the TM7a-like phylotype (Table 1). Through qPCR we determined the concentration of the three phylogenetic group levels Bacteria, the TM7, and TM7a-like in three sludge sites (S1, S2, and S3) from eight collection dates between August 2007 and January 2010, except for S1 in March 2008 for the lack of genomic DNA (Figure 2A), hence a total of 23 qPCR samples. While Bacteria and TM7 were detected in all 23 (100%) sludge samples, TM7a-like was detected in 4/23 (17.4%) samples The qPCR values for TM7a-like quantification fell below detection limit in 19/23 (83%) samples, partially explained by known limitations of qPCR assays [22]. This was supported by sequences of TM7a-like recovered by cloning DNA amplicons from sludge sample S1 in June 2009. The conservative qPCR analyses shown included only values with 3 qPCR cycles above the negative controls.

**Table 1:** 16S rDNA gene amplification parameters for qPCR assay from activated wastewater.

qPCR Assay Targets^a^	Amplification Parameters				
	Amplification efficiency ± SD	Slope ± SD	y intercept ± SD	R^2^	Standard Range
General Bacteria	1.02±0.025	−3.29±0.056	37.7±0.80	>.98	10^3^–10^8^
Division TM7	0.96±. 0.019	−3.44±0.051	36.0±0.48	>.99	10^2^–10^7^
TM7a-like	0.92±0.020	−3.53±0.056	35.5±0.65	>.99	10^1^–10^6^

a Each assay target consisted of three triplicate runs, total sample population (n = 9).

Concentration of Bacteria, TM7, and TM7a-like in cells ml^−1^ of sludge were dynamic over time based on qPCR values (Figure 2A). Bacteria concentration ranged from 1.30×10^7^ (March 2008) to 1.28×10^9^ cells ml^−1^ (September 2009) with an overall average of 3.47×10^8^±8.00×10^7^ (mean ± s.e.m.; n = 23) cells ml^−1^ from all samples combined (Figure 2A1). TM7 counts ranged from 3.30×10^5^ (June 2009) to 5.84×10^7^ cells ml^−1^ (September 2009) with an average of 1.09×10^7^±3.17×10^6^ cells ml^−1^ (n = 23) for all samples combined (Figure 2A2). TM7a-like counts ranged from 4.02×10^3^ (March 2008) to 7.84×10^4^ cells ml^−1^ (August 2007) with an average of 2.84×10^4^±1.74×10^4^ cells ml^−1^ (n = 4) for all samples (Figure 2A3).

The average relative abundance of TM7 (average TM7/average Bacteria) was 3.02%±3.80% (n = 23) with the highest value detected in S1 in May 2008 at 6.87%±3.72% (n = 9). For TM7a-like, the average relative abundance to Bacteria (average TM7a-like/average Bacteria) was 0.01%±0.01% (n = 4) and to TM7 (average TM7a-like/average TM7) was 0.44%±0.18% (n = 4). The highest relative abundance of TM7a-like to Bacteria was detected in S2 in August 2007 at 0.017%±0.025% (n = 4) and to TM7 in S2 in April 2008 at 1.07%±0.15% (n = 3).

Two-way ANOVA comparisons of TM7 concentration (cell ml^−1^, qPCR) for each time point between each of the three sampling sites at 95% confidence limit showed that S1 was statistically significantly higher than S2 (p = 0.0001, t = 4.640, n = 18) in August 2007, than S3 (p = 0.01, t = 3.643, n = 18) in May 2008, and than S2 (p = 0.0001, t = 8.178, n = 14) and S3 (p = 0.01, t = 3.860, n = 16) in October 2009 (Figure 2A2).

Because many qPCR measurements for TM7a-like were below detectable levels, we used One-Way ANOVA with Bonferroni multiple comparisons to determine if the average qPCR values of the TM7a phylotype was statistically higher 1) at any particular time of the year for each of the three sites, and 2) in any one of the three sampling sites as compared to the other two sites. Environmental TM7a populations in S2 in August 2007 were statistically significantly higher than all other S2 counts (March 2008, n = 6, p = 0.05, t = 7.538 and April 2008, n = 3, p = 0.05, t = 7.400) and S3 counts (October 2009, n = 6, t = 5.389, p = 0.05) at 95% confidence.

### Fluorescence *In Situ* Hybridization

Whole-cell quantification through Fluorescence *In Situ* Hybridization (FISH) detected TM7 and environmental TM7a in all analyzed sludge samples. FISH cell counts were performed in nine samples collected in June, August, and September 2010 at the same three sites as qPCR samples (Figure 2B). Concentration of FISH-labeled TM7 cells (Figure 2B2) ranged from 6.94×10^5^ to 7.22×10^6^ cells ml^−1^ (September 2010) and averaged 2.16×10^6^±7.65×10^5^ (n = 9) cells ml^−1^ for all sample sites combined. TM7a-like cell concentrations (Figure 2B3) ranged from 1.08×10^4^ (June 2010) to 1.84×10^5^ cells mL^−1^ (September 2010), and averaged 6.63×10^4^±1.97×10^4^ cells mL^−1^ (n = 8) for all samples.

Statistical analysis of FISH counts showed that in September 2010, TM7 populations were significantly higher in S1 (n = 10, t = 3.259 p = 0.05, Two-Way ANOVA) and S2 (n = 10, t = 4.457 p = 0.001, Two-Way ANOVA) than S3 (Figure 2B2). No other significant differences in mean populations were determined. In contrast, the relative abundances of TM7 and environmental TM7a of the Bacteria were 1.05%±0.23% (n = 9) and 0.034%±0.01% (n = 9), respectively, while TM7a relative abundance of TM7 was 7.76%±3.07% (n = 9).

**Figure 3:**
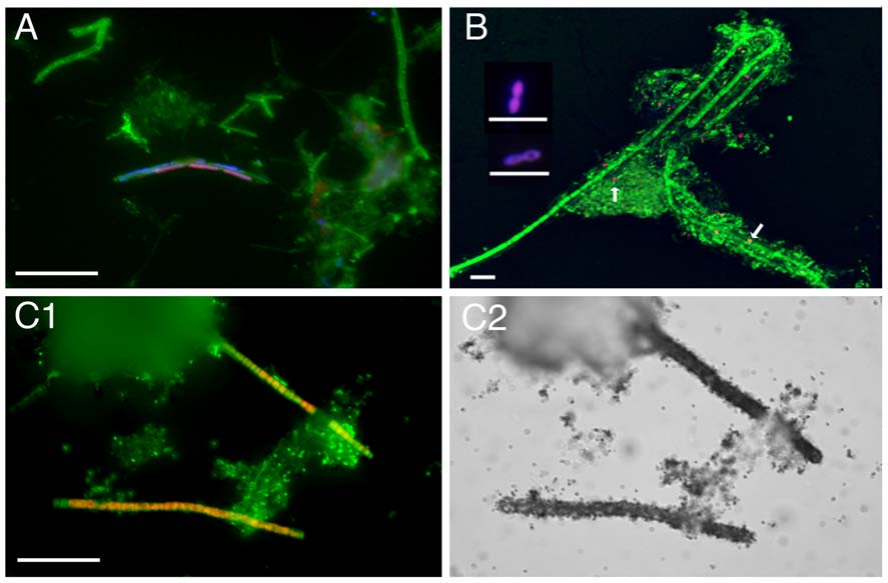
Micrographs depicting TM7 (red) and TM7a-like (blue) in the total microbial community (green) of activated sludge samples through (A–B) FISH and STARFISH (C1–C2). (**A**) TM7a-like short filamentous cells with three segments labeled with both TM7 and TM7a probes. (**B**) TM7 and TM7a-like cells (latter marked with arrows) as coccobacilli and cocci; two insets show TM7a diplo-bacillus morphologies commonly found in sludge. (**C**) TM7 long filamentous cells (**C1**, red-orange) taking up a mixture of dissolved tritiated amino acids (**C2**, bright field) through micro-autoradiography (STARFISH) assay, suggesting TM7 are metabolic active in wastewater. Scale bars = 5 µm.

Microscopic FISH visualization was also used to characterize morphological features of TM7 and TM7a-like cells in sludge samples (Figure 3). TM7 cells ranged from short rods (2.5×0.5 µm) and cocco-bacilli (2.0×0.7 µm) to long (up to 40.0 µm×1.2 µm) filaments (Figure 3A). TM7a-like cells, however, consisted mostly of diplococci and short rods (Figure 3B), found either isolated or within cell aggregates.

### Substrate-Tracking Autoradiography with Fluorescence *In Situ* Hybridization

Substrate-Tracking Autoradiography Fluorescence *In Situ* Hybridization (STARFISH) [23] assays showed TM7 cells were metabolically active prior to fixation (Figure 3C). All TM7 cells fluorescently labeled through STARFISH (Figure 3C1) were able to take up a mixture of 15 tritiated-amino acids and were heavily labeled through autoradiography (Figure 3C2). We were not able to detect cells labeled with the TM7a-1033 fluorescent probe in our STARFISH experiments in part due to the labor intensity of the STARFISH method, the low relative abundance of those cells, and the high concentration of detritus interfering with cell visibility.

## Discussion

This survey identified a continuous environmental source for a TM7 bacterium with 16S rDNA sequence highly homologous to a human-associated phylotype. In fact, our TM7a-like has higher sequence identity to the human-associated TM7a than other TM7 found elsewhere in the human body hitherto. We considered an *environment* any sample site outside an animal host. It is well known the microbial consortia of wastewater is complex and include prokaryotes from host organisms, including humans. Our goal, nonetheless, was to identify a source of potentially viable model microorganisms of easier access than humans, independent of the environment. The TM7a-like was readily detected in all seven clone libraries over this two-year project and FISH hybridization confirmed the TM7a-like cells had detectable levels of rRNA prior to fixation.

For an uncultured bacterial model to be practical, the bacterium must be prevalent and easily detectable. Our sludge clone AAWS56C formed a monophyletic group with other human-associated phylotypes from oral plaque and skin samples (Figure 1, enclosure *TM7a Group*). To our surprise, three other environmental TM7 phylotypes from independent studies shared class-level (92.2–93.7%) homology with human-associated phylotypes. In Enclosure-I, EF515301 “microbial fuel cell” shared 92.9% identity with Vaginal epithelial DQ666092; in Enclosure-II, GQ264495 “waste site” shared 93.7% identity with human “subgingival crevice SBG2” AY144354, and in Enclosure-IV, FJ671754 “beef cattle feedlot” shared 92.2% identity to “subgingival plaque” AY005446. A fourth phylotype (AJ318200 “waste-gas biofilter”) shared genus-level (95.2%) homology with human “subgingival plaque” GU227158 (Figure 1, enclosure III). These independent associations suggest that additional environmental and human-associated relationships within the Candidate Division TM7 can be explored.

We present one of the most comprehensive phylogenies of the TM7 Candidate Division to date. A previous TM7 phylogeny of similar scale [10] suggested human-associated TM7 formed a monophyletic group. With nearly ten times more TM7 sequences available for our analysis, it appears that human-associated TM7 cluster with environmental and other animal hosts, although, all human-associated TM7 phylotypes clustered within subdivision 2. Phylogeny unveiled additional prospective environmental sources of TM7 models to different human oral and vaginal phylotypes.

Community statistical analyses supported the phylogenetic similarity between wastewater and human phylotypes within the TM7a Group even though environmental TM7 were significantly different from human-associated TM7 when all phylotypes in Figure 1 were clustered by community type. Statistical analyses using all phylotypes in Figure 1 supported the higher similarity between TM7 from humans and TM7 from other animals than the TM7 from either one of those two communities compared to TM7 from environmental sites. In other words, similarity of TM7 among the three communities can be summarized based on the relationship E<A<H. However, no significant statistical difference was observed among the eight phylotypes within the TM7a Group enclosure. Under standard approaches to phylogenetic tree building the eight phylotypes in the TM7a Group would be depicted as a single phylotype, but all eight sequences were purposely included to reinforce the diversity of TM7 from environmental sites with high identity to TM7 in humans.

Understanding the model organism population abundances and distributions may help one maximize retrieval from samples. The relative abundance of TM7 and TM7a from qPCR counts over the entire course of the study, for instance, provided insights for enriched samples with these phylotypes. Statistical analysis of qPCR values indicated that S1 had the overall highest concentrations of TM7 and S2 the highest relative abundance of TM7a-like. Even though the overall relative abundance of TM7 cells in our sludge samples represented a small fraction of the total Bacteria, it was 5 times greater than TM7 in healthy human oral sites (0.21%±0.05%) and ∼2 times as the diseased sites with mild periodontitis (0.54%±0.10%) based on FISH counts [14], making sludge more amenable for sensitive experimentation with TM7. None of the human-associated TM7a group phylotypes have yet been quantified for comparison to our environmental model FISH counts, but cell morphologies of our sludge TM7 were similar to other environmental [10] and human oral [24] studies.

Future characterization of the TM7a-like through single-cell genomic amplification [20], nutrient uptake requirements [25], and probe protein-coding RNA [26] can advance its functional homology to human-associated TM7. Extrapolation from this TM7 study can potentially render many other human-associated models for uncultured Bacteria and Archaea.

Humans have been referred to as “supraorganisms” [27] whose total genetics constitute a mixture of human and microbial sources. The vast majority of those microbial genes reside within parts of the human body difficult to access such as the crevices of the gut. If those same genes are more accessible elsewhere, their biological activities can be rendered similar to the way we have used cultivable bacteria as model organisms.

## Materials and Methods

### Ethics

Protocols for collecting bacteria on human skin and oral surfaces were approved by the San Jose State University Institutional Review Board committee through Protocol # F0904009 prior to collection. Written consent forms were provided to and signed by each patient prior to sampling and all human samples were collected and processed at SJSU through that same IRB protocol. A copy of the consent form has been provided to the editor of the journal. Collection of sludge samples was approved verbally or through email prior to every visit to the San Jose - Santa Clara County Water Pollution Control Plant. A staff member of the plant accompanied one of our researchers during the collection.

### Environmental Site Survey

We surveyed numerous environmental sites with similar characteristics to environments where TM7 had been previously detected [10] to locate a TM7 reservoir. Sites with old forest and garden soils, rhizosphere, seawater, and sludge were analyzed through 16S rDNA pCR, cloning, and sequencing. Aerobic sludge samples were selected due to the presence of human-associated TM7 homologues, among other highly diverse TM7 phylotypes. All procedures described below apply to activated wastewater sludge samples only.

### Sample Collection, Preparation, and DNA Extraction

A total of 23 samples, each with 200 ml of activated sludge, were collected in sterile Whirl-Pak bags (Nasco, Fort Atkinson, WI) from three sites at the San Jose-Santa Clara County Water Pollution Control Plant between August 2007 and January 2010. Samples were homogenized by shaking and for a few of the sludge samples, 40 ml were aliquoted to a sterile 50 ml conical tube and fixed in 50% methanol. All sample aliquots were immediately stored in ice and transported to the lab (20 min transit time) for processing. Once in the lab, live samples were immediately processed for genomic DNA extraction and Substrate-Tracking Auto-Radiography Fluorescence *In Situ* Hybridization (STARFISH) [23], whereas fixed samples were prepared for DAPI and FISH counts as explained below. Genomic DNA was extracted from all 23 sludge samples following a bead beater half-lysis protocol [28], [29]. First, 30 g of neat sample weighed in sterile 50 ml conical tubes were centrifuged at 9,000 g for 15 min at 4°C. The supernatant was discarded and 0.25 g of the pellet was used for genomic DNA extraction.

### PCR amplification, Cloning, and Sequencing

Each genomic DNA extraction was first screened for TM7 via PCR amplification of the 16S rDNA gene (∼1,200 bp) with the broad-range forward primer BAC-8F and the TM7-1177R (Table S1, Supporting online information) with 100× diluted DNA. Each PCR reaction consisted of 1× PCR Buffer B (Fisher Scientific, Waltham, Massachusetts), 1.5 mM MgCl2, 0.2 mM of each deoxynucleoside triphosphate, 0.2 pmol of each forward and reverse primer, 1.25×10−2 units of AmpliTaq DNA polymerase (Applied Biosystems, Foster City, California), and 3 to 30 ng of nucleic acid. Screening PCR cycling conditions included a 3 min 96°C hot start, followed by 40 cycles at 94°C for 1 min denaturation, 61°C for 1 min annealing, and 72°C for 1 min elongation, with a final elongation step at 72°C for 3 min. PCR positive samples were amplified *de novo* in a 50 µl PCR reaction volume and 30 PCR cycles to minimize PCR bias [30]. PCR amplicons (1,200 bp) were cleaned in a 2% agarose E-gel and E-gel CloneWell Safe-Imager real-time transilluminator (Invitrogen, Carlsbad, CA), then cloned in Invitrogen pCR2.1-TOPO vector, and transformed into One Shot TOP10 competent cells following Invitrogen protocols for blue-white screening. Plasmids were purified from 303 clones with QIAprep Spin Miniprep Kit (Qiagen, Valencia, CA) and sequenced with M13F primer by Sequetech, Mountain View, CA.

### DNA Sequence Analysis

All 16S rDNA partial sequences generated from our clone libraries were cleaned of poor quality bases and PCR primers in CodonCode Aligner v 3.0.2 software (CodonCode Corporation, Dedham, MA), then checked for TM7 identity through Basic Local Alignment Search Tool (BLAST) [31] in GenBank [32]. Sequences with ≥98.5% identity to a human-associated TM7 based on BLAST as well as unique TM7 clones (≤98.5% to any clone in GenBank) were fully sequenced. Each consensus sequence (∼1,140 bp) was screened for chimera using Chimera Check in Bellerophon v 3.0 before being aligned using SINA Webaligner (http://www.arb-silva.de/aligner) [33].

### Phylogenetic Tree

Aligned sequences were imported into ARB database software package [34] and compared to 541 TM7 reference sequences collected from the SILVA rRNA project [33] and Genebank with length ≥1,200 bp. All sequences were manually aligned based on 16S rDNA primary and secondary structures. A 1,052 unambiguous column filter was generated. A total of 255 Candidate Division TM7 sequences were used in the final phylogenetic analysis. The tree contains 208 unique OTUs based on DOTUR analysis [35] at a 99% identity threshold (99% was used here instead of 98.5% due to DOTUR automatic rounding). A neighbor-joining tree with Felsenstein correction was generated using PHYLIP interference package (28). Tree topography was tested by bootstrap re-sampling analysis of 1,000 replications. thresholds for approximate species level classification. Bootstrap values ≥50% were included. Tree sequence metadata were manually curated, annotated, and the interactive tree online was generated with jsPhyloSVG [36]. Community statistical analyses of the TM7 diversity in Figure 1 and well for the TM7a Group lineage were performed with UniFrac online computational tools [21].

### Quantitative PCR (qPCR)

Samples with confirmed TM7 sequences were processed through qPCR to quantify the total Bacteria, the total TM7, and the TM7a-like 16 rDNA gene copy number in 23 sludge samples. Quantification of each of the three phylogenetic groups was performed in separate qPCR runs, each run with its own standard curve in triplicates, and each full assay was repeated 2–4 times on an ABI 7300 instrument (Applied Biosystems, Foster City, CA). TM7a group-specific primers (ENV-TM7a-1112R, TM7a-997F) and probe (TM7a-1033) were designed for this work (Table S1) and validated against target plasmids DNA with 1–4 mismatches at primer or probe target site. TaqMan primer pair combinations are listed in Table S2 (Supporting Information).

The gene copy number from Bacteria, TM7 and TM7a-like qPCR reactions were converted to cells ml^−1^ of neat activated wastewater based on these four corrections: i) corrections for nucleic acid dilutions used for PCR amplification, ii) corrections for target DNA concentration, iii) corrections for changes in volume during activated wastewater processing, and iv) an estimated 3.5 average rDNA gene copy number per cell for bacteria quantification [37] or an estimated 2.0 average rDNA gene copy number per cell for TM7 and for TM7a-like quantification. The estimated average 16S rDNA gene copy number per cell of TM7 and per cell of TM7a-like was determined based on complete and partial genome assemblages belonging to each respective group and deposited in public databases [20], [38].

Each qPCR reaction mixture to quantify total Bacteria, TM7, and TM7a-like 16S rDNA gene copy numbers had a final volume of 20 µl made of 10 µl Taqman Universal PCR Master Mix (Applied Biosystems, Foster City, California), an additional 2.5×10^−2^ units of AmpliTaq DNA polymerase (Applied Biosystems, Foster City, California), and primers and fluorescent probe for each of the three groups as follows: i) Bacteria qPCR: 1.8 pmol of primer BAC 8F, 3.6 pmol of primer BAC 515R, and 0.1 pmol of each Taqman probe BAC 338I, BAC 338II, and BAC 338III; ii) TM7 qPCR: 0.9 pmol of each primer TM7 910F and TM7 1177R and 0.1 pmol of Taqman probe TM7-1093; and iii) TM7a-like qPCR: 0.9 pmol of primer TM7a-997F, 0.9 pmol of primer ENV-TM7a-1112R, and 0.1 pmol of TM7a-1033 probe. qPCR cycling conditions included an initial denaturation at 95°C for 10 min followed by 50 cycles at 95°C for 30 sec, annealing temperature for Bacteria, TM7, and TM7a-like listed in Table S2 for 30 sec, and 72°C for 30 sec. Fluorescence levels for all qPCR runs were read at the end of each 72°C elongation step.

### Fluorescence *In Situ* Hybridization (FISH) and Fluorescence Microscopy

First, we determined sample volumes per well of Teflon-coated slides to generate an average distribution of 100–500 cells per microscopic field of view through DAPI-stained cell counts of the 50% methanol-fixed sludge samples under epifluorescence microscopy as previously explained [39]. Fixed sludge samples were then transferred to Teflon-coated slides with ADD-cell adherence coating (Fisher Scientific, Waltham, Mass). FISH conditions for TM7-905 probe has been published [39]. The TM7a-1033 FISH probe (Table S1) was designed in ARB for this work and empirically validated at a single nucleotide discrimination via catFISH [40], a method that relies on expression of heterologous TM7 rRNA targets of varying number of nucleotide mismatches to the TM7a group probe. Hybridization conditions were optimized until only cells with perfect-match rRNA targets were visible through FISH. FISH used published probes and protocols for TM7 [10], [39] and our probe TM7a-1033 was catFISH [39] validated at 30% formamide. FISH imaging and counts used a Zeiss Axioscope-A.1 microscope, Hammamatsu camera, and AxioVision 4.7 software. Zeiss filters #49, #43, and #50 detected DAPI, Cy3, and Cy5, respectively.

### Substrate-Tracking AutoRadiography with Fluorescence *In Situ* Hybridization (STARFISH)

STARFISH detects single cell capacity to take up a radioactively labeled nutrient by combining FISH with autoradiography [24], [25]. We used STARFISH to determine the metabolic activity of TM7 by adding trace amounts (∼5 nM) of a mixture of 15 tritiated amino acids (American Radiolabeled Chemicals, Inc, St Louis, MO) to the sludge and incubating at ambient temperature and aerobic conditions for one hour before fixing the samples in 50% methanol. Fluorescence imaging was the same as for FISH above with the additional fourth detection with transmitted light for the autoradiography signal.

### Lab Member Skin and Oral Screening

Potential contamination of sludge samples with TM7 bacterial DNA associated with our lab members was tested by PCR screening with primers BAC-8F and HUM-TM7a-1112R, cloning, and sequencing 174 clones. From each of 10 individuals, we collected seven samples: six skin samples and one oral cavity sample (following approved guidelines on IRB protocol F0904009). Skin samples from the antecubital fossa, hypothenar palm, ear, umbilicus, and scalp were collected with a sterile cotton swab moist in sterile MilliQ water, whereas tooth scrapings were collected with a sterile plastic tip by scrapping several tooth surfaces. Each sample was placed in 100 ml sterile MilliQ water, pelleted at 5,000 g for 10 min at room temperature, and resuspended in 180 µl enzymatic lysis buffer (20 mM Tris-HCl pH 8.0, 2 mM sodium EDTA, 1.2% Triton X-100, 20 mg/ml Lysozyme). Genomic DNA was extracted using the DNeasy Blood and Tissue Kit following the Gram-Positive Bacteria Protocol (Qiagen, Valencia, CA). Conditions for PCR, cloning and sequence analysis were the same as those described for sludge samples above, except that human samples did not require dilution and the PCR annealing temperature was 58°C.

## Supporting Information

Table S1 (DOCX)

Table S2 (DOCX)

Figure S1 Nucleotide-base comparison of aligned 16S rDNA gene sequences obtained from this study and reference sequences in the (*) TM7a Group in Figure1. Symbols above some bases indicate single nucleotide polymorphism (SNP) between the Activated Wastewater HM208134 (TM7a-like) clone and (+) Antecubital fossa HM269723 and Subgingival crevice SBG3 sequences, (Δ) all other members of the TM7a Group, (◊) and all analyzed sequences. This comparison was performed using CLC Main Workbench 5.
(PDF)
