# Supplementary material for: In Search of an Uncultured Human-Associated TM7 Bacterium in the Environment
Source: PLoS One. 2011 Jun 20;6(6):e21280. doi: 10.1371/journal.pone.0021280 (PMC3118805; doi:10.1371/journal.pone.0021280)
Supplement: Table S1 — (DOCX) [file pone.0021280.s001.docx]

Table S1

| **Table S1. Primers and probes for Quantitative PCR (qPCR) and Fluorescence *In Situ* Hybridization (FISH) targeting the 16S rDNA gene.** | | | | | | | |
| --- | --- | --- | --- | --- | --- | --- | --- |
| Name | Function | Target | Sequence (5'-3') | T_m_  (^o^C)  50 mM Na^+^ | % GC | Label | Reference |
| **Primers** |  |  |  |  |  |  |  |
| BAC-8F | Cloning, qPCR | Bacteria | AGAGTTTGATCMTGGCTCAG | 49.7-51.8 | 45.0-50.0 | ---------- | 14 |
| BAC-515R | qPCR | Bacteria | KACCGCGGCKGCTGGCA | 54.3-59.1 | 71.0-82.0 | ---------- | 14 |
| TM7-910F | qPCR | TM7 | CATAAAGGAATTGACGGGGAC | 52.4 | 48.0 | ---------- | 14 |
| TM7-1177R | Cloning, qPCR | TM7 | GACCTGACATCATCCCCTCCTTCC | 60.8 | 58.0 | ---------- | 14 |
| TM7a-997F | Cloning, qPCR | TM7a Group | TCCCGAGAAGATTTACG | 44.6 | 47.0 | ---------- | This work |
| HUM-TM7a-1112R | Cloning | Human TM7a | ACAACTAGACACAAGGG | 44.6 | 47.0 | ---------- | This work |
| ENV-TM7a-1112R | Cloning, qPCR | TM7a-like | TCAACTATTCACAAGGG | 42.2 | 41.0 | ---------- | This work |
| **Probes** |  |  |  |  |  |  |  |
| TM7-1093 | qPCR | TM7 | AGTCCATCAACGAGCGCAACC | 56.3 | 57.0 | VIC-BHQ | 14 |
| TM7a-1033 | qPCR | TM7a Group | ATCTGTCACCGAGTTCCA | 48.0 | 50.0 | FAM- BHQ | This work |
| BAC-338I | qPCR | Bacteria | GCTGCCTCCCGTAGGAGT | 54.9 | 67.0 | FAM-TAMRA | 14 |
| BAC-338II | qPCR | Bacteria | GCAGCCACCCGTAGGTGT | 54.9 | 67.0 | FAM- BHQ | 14 |
| BAC-338III | qPCR | Bacteria | GCTGCCACCCGTAGGTGT | 54.9 | 67.0 | FAM- BHQ | 14 |
| TM7-905 | FISH | TM7 | CCGTCAATTCCTTTATGTTTTA | 47.4 | 32.0 | CY5 | 8 |
| TM7a-1033 | FISH | TM7a Group | ATCTGTCACCGAGTTCCA | 48.0 | 50.0 | CY3 | This work |
