# Supplementary material for: In Search of an Uncultured Human-Associated TM7 Bacterium in the Environment
Source: PLoS One. 2011 Jun 20;6(6):e21280. doi: 10.1371/journal.pone.0021280 (PMC3118805; doi:10.1371/journal.pone.0021280)
Supplement: Table S2 — (DOCX) [file pone.0021280.s002.docx]

Table S2

| **Table S2. qPCR primer pairs used in assays targeting Bacteria, TM7, and TM7a-like with respective amplicon size and annealing temperature(s).** | | | | |
| --- | --- | --- | --- | --- |
| **Target Group** | **Forward Primer** | **Reverse Primer** | **Amplicon Size (bp)** | **Annealing temp. (^0^C)** |
| Bacteria | BAC-8F | ^a^BAC-515R | 507 | 55, 60, 64^a^ |
| Division TM7 | TM7-910F | TM7-1177R | 267 | 61 |
| TM7a Group | TM7a-997F | ENV-TM7a-1112R | 115 | 57 |
| ^a^Primer optimized to these three annealing temperatures due to degenerative bases as previously described [[14](#_ENREF_14)]. | | | | |
