# Supplementary material for: In Search of an Uncultured Human-Associated TM7 Bacterium in the Environment
Source: PLoS One. 2011 Jun 20;6(6):e21280. doi: 10.1371/journal.pone.0021280 (PMC3118805; doi:10.1371/journal.pone.0021280)
Supplement: Figure S1 — Nucleotide-base comparison of aligned 16S rDNA gene sequences obtained from this study and reference sequences in the (*) TM7a Group in Figure1. Symbols above some bases indicate single nucleotide polymorphism (SNP) between the Activated Wastewater HM208134 (TM7a-like) clone and (+) Antecubital fossa HM269723 and Subgingival crevice SBG3 sequences, (Δ) all other members of the TM7a Group, (◊) and all analyzed sequences. This comparison was performed using CLC Main Workbench 5. (PDF) [file pone.0021280.s003.pdf]

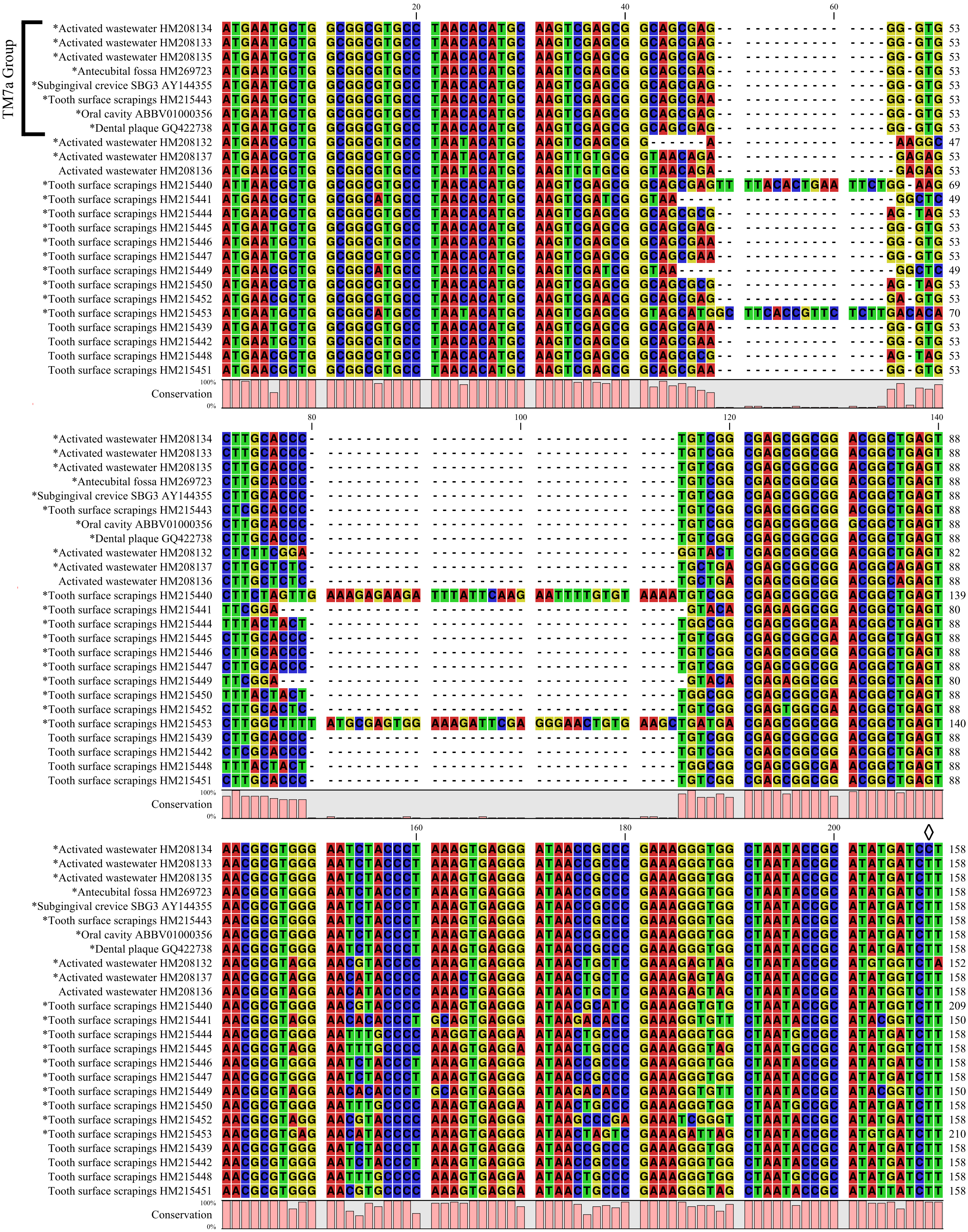

TM7a Group
*Activated wastewater HM208134
*Activated wastewater HM208133
*Activated wastewater HM208135
*Antecubital fossa HM269723
*Subgingival crevice SBG3 AY144355
*Tooth surface scrapings HM215443
*Oral cavity ABBV01000356
*Dental plaque GQ422738
*Activated wastewater HM208132
*Activated wastewater HM208137
Activated wastewater HM208136
*Tooth surface scrapings HM215440
*Tooth surface scrapings HM215441
*Tooth surface scrapings HM215444
*Tooth surface scrapings HM215445
*Tooth surface scrapings HM215446
*Tooth surface scrapings HM215447
*Tooth surface scrapings HM215449
*Tooth surface scrapings HM215450
*Tooth surface scrapings HM215452
*Tooth surface scrapings HM215453
Tooth surface scrapings HM215439
Tooth surface scrapings HM215442
Tooth surface scrapings HM215448
Tooth surface scrapings HM215451
Conservation
100%
0%

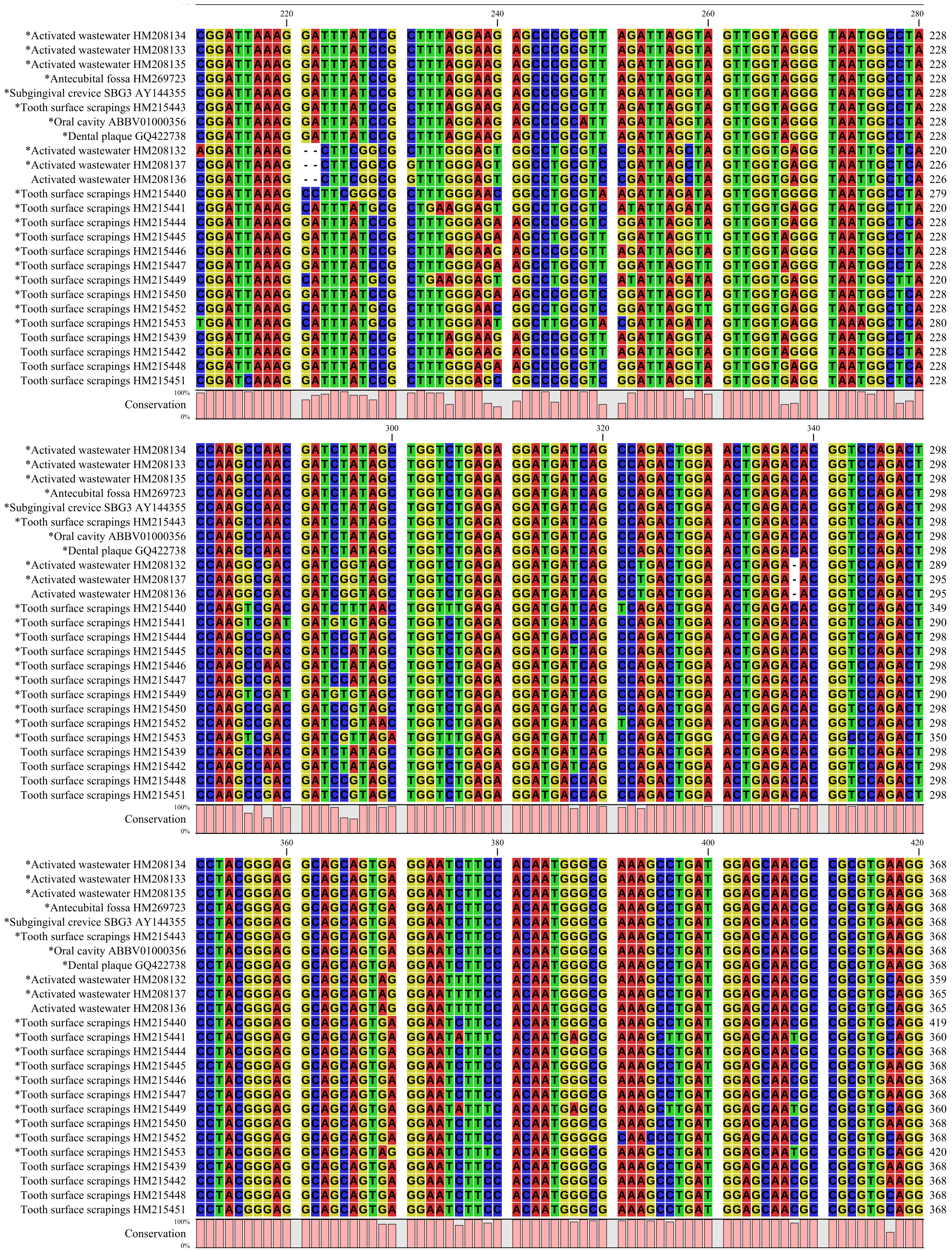

220
240
260
280
*Activated wastewater HM208134
*Activated wastewater HM208133
*Activated wastewater HM208135
*Antecubital fossa HM269723
*Subgingival crevice SBG3 AY144355
*Tooth surface scrapings HM215443
*Oral cavity ABBV01000356
*Dental plaque GQ422738
*Activated wastewater HM208132
*Activated wastewater HM208137
Activated wastewater HM208136
*Tooth surface scrapings HM215440
*Tooth surface scrapings HM215441
*Tooth surface scrapings HM215444
*Tooth surface scrapings HM215445
*Tooth surface scrapings HM215446
*Tooth surface scrapings HM215447
*Tooth surface scrapings HM215449
*Tooth surface scrapings HM215450
*Tooth surface scrapings HM215452
*Tooth surface scrapings HM215453
Tooth surface scrapings HM215439
Tooth surface scrapings HM215442
Tooth surface scrapings HM215448
Tooth surface scrapings HM215451
Conservation
100%
0%
300
320
340
360
380
400
420

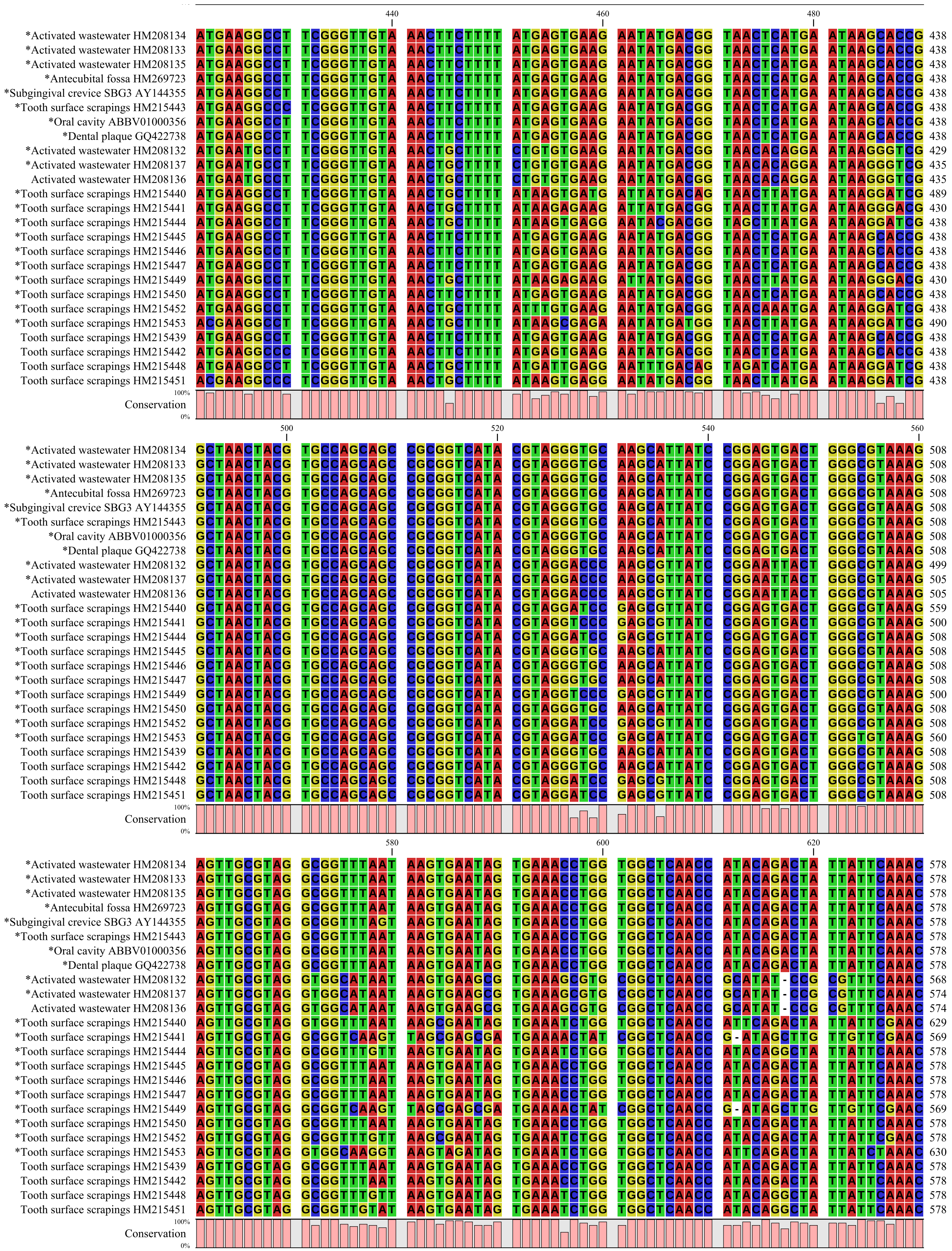

440
460
480
*Activated wastewater HM208134
*Activated wastewater HM208133
*Activated wastewater HM208135
*Antecubital fossa HM269723
*Subgingival crevice SBG3 AY144355
*Tooth surface scrapings HM215443
*Oral cavity ABBV01000356
*Dental plaque GQ422738
*Activated wastewater HM208132
*Activated wastewater HM208137
Activated wastewater HM208136
*Tooth surface scrapings HM215440
*Tooth surface scrapings HM215441
*Tooth surface scrapings HM215444
*Tooth surface scrapings HM215445
*Tooth surface scrapings HM215446
*Tooth surface scrapings HM215447
*Tooth surface scrapings HM215449
*Tooth surface scrapings HM215450
*Tooth surface scrapings HM215452
*Tooth surface scrapings HM215453
Tooth surface scrapings HM215439
Tooth surface scrapings HM215442
Tooth surface scrapings HM215448
Tooth surface scrapings HM215451
438 438 438 438 438 438 438 438 429 435 435 489 430 438 438 438 438 430 438 438 490 438 438 438 438
Conservation
100%
0%
500
520
540
560
508 508 508 508 508 508 508 508 499 505 505 559 500 508 508 508 508 500 508 508 560 508 508 508 508
580
600
620
578 578 578 578 578 578 578 578 568 574 574 629 569 578 578 578 578 569 578 578 630 578 578 578 578

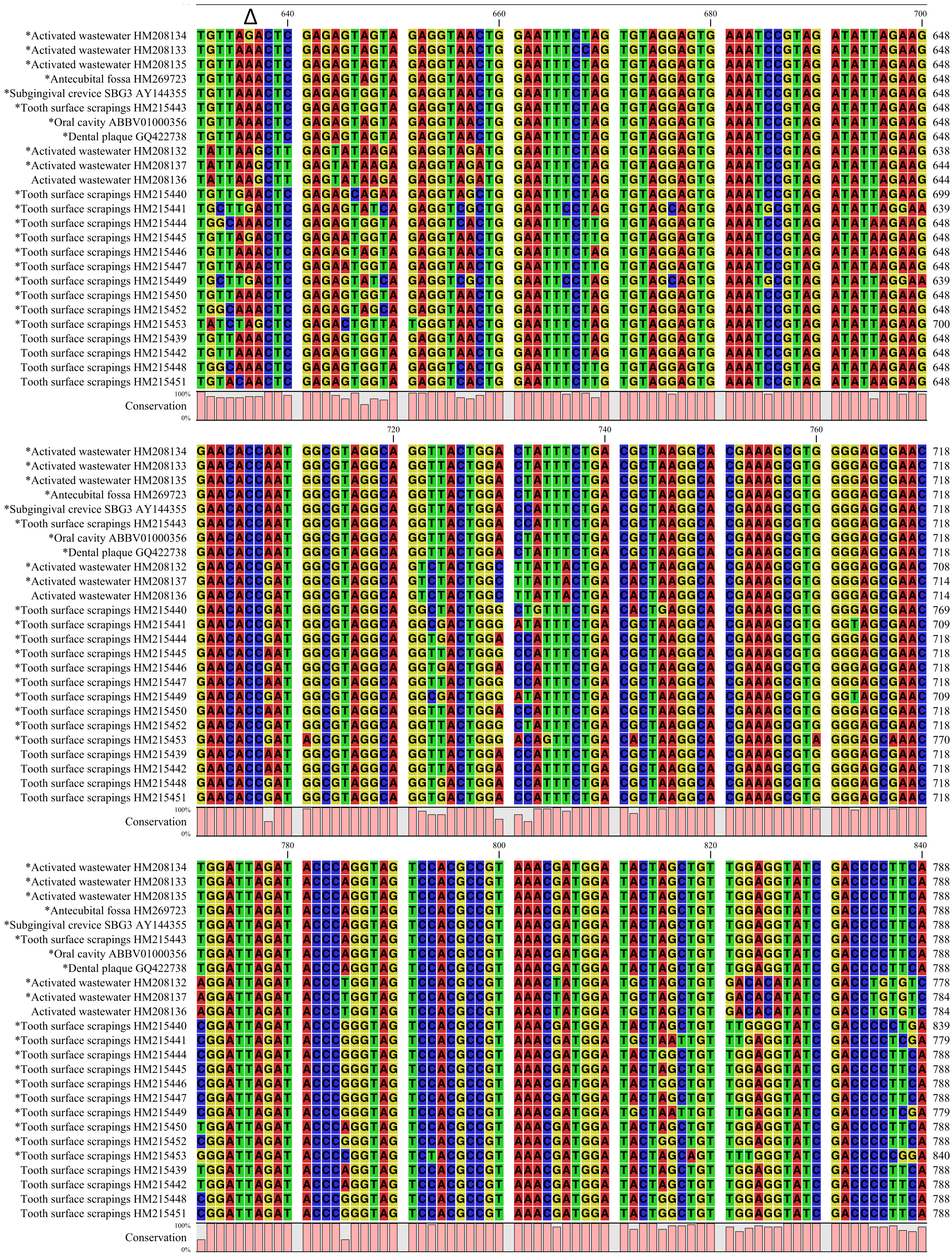
Δ
*Activated wastewater HM208134
*Activated wastewater HM208133
*Activated wastewater HM208135
*Antecubital fossa HM269723
*Subgingival crevice SBG3 AY144355
*Tooth surface scrapings HM215443
*Oral cavity ABBV01000356
*Dental plaque GQ422738
*Activated wastewater HM208132
*Activated wastewater HM208137
Activated wastewater HM208136
*Tooth surface scrapings HM215440
*Tooth surface scrapings HM215441
*Tooth surface scrapings HM215444
*Tooth surface scrapings HM215445
*Tooth surface scrapings HM215446
*Tooth surface scrapings HM215447
*Tooth surface scrapings HM215449
*Tooth surface scrapings HM215450
*Tooth surface scrapings HM215452
*Tooth surface scrapings HM215453
Tooth surface scrapings HM215439
Tooth surface scrapings HM215442
Tooth surface scrapings HM215448
Tooth surface scrapings HM215451
Conservation
100%
0%
640
660
680
700
720
740
760
780
800
820
840

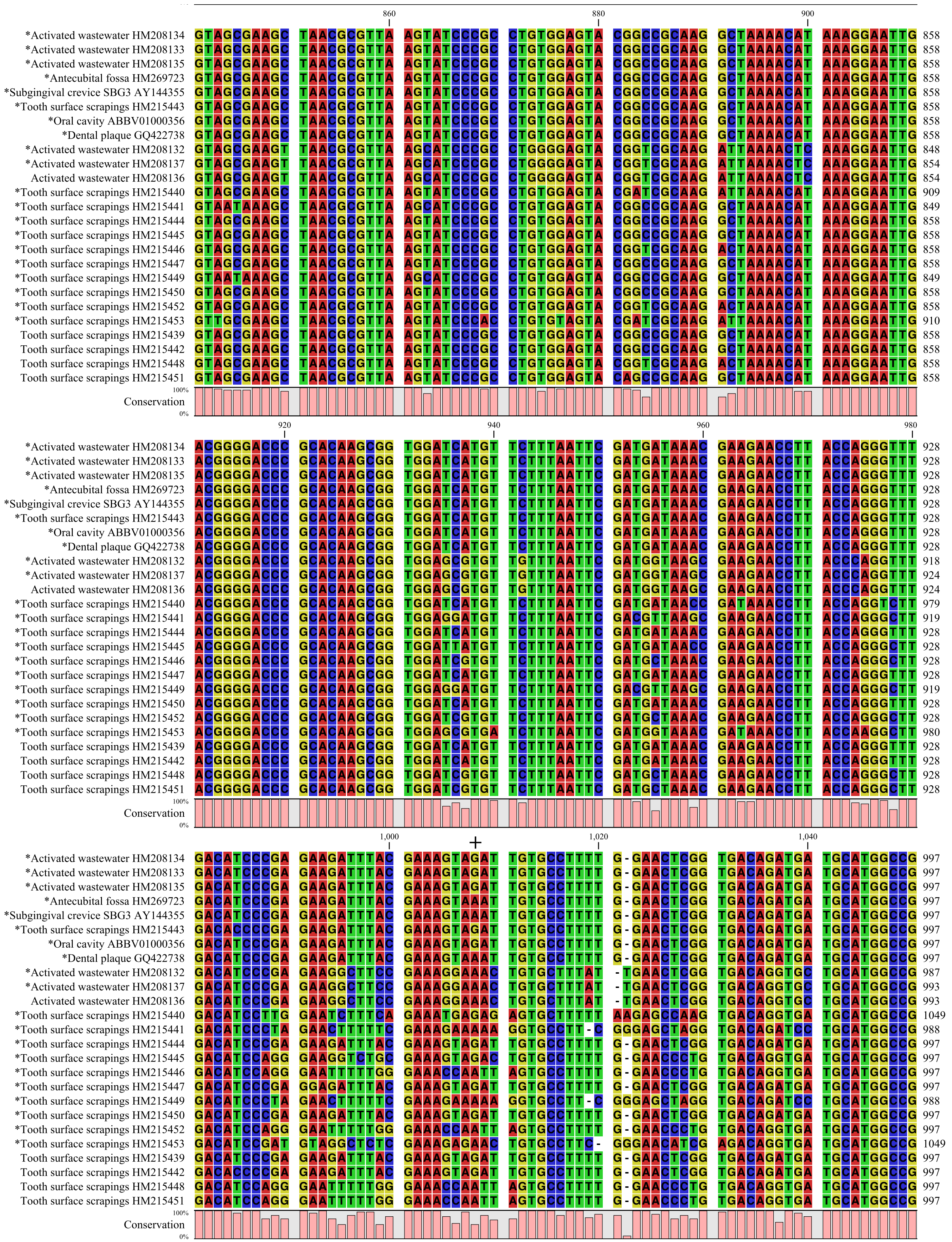

860
880
900
920
940
960
980
1,000
1,020
1,040
*Activated wastewater HM208134
*Activated wastewater HM208133
*Activated wastewater HM208135
*Antecubital fossa HM269723
*Subgingival crevice SBG3 AY144355
*Tooth surface scrapings HM215443
*Oral cavity ABBV01000356
*Dental plaque GQ422738
*Activated wastewater HM208132
*Activated wastewater HM208137
Activated wastewater HM208136
*Tooth surface scrapings HM215440
*Tooth surface scrapings HM215441
*Tooth surface scrapings HM215444
*Tooth surface scrapings HM215445
*Tooth surface scrapings HM215446
*Tooth surface scrapings HM215447
*Tooth surface scrapings HM215449
*Tooth surface scrapings HM215450
*Tooth surface scrapings HM215452
*Tooth surface scrapings HM215453
Tooth surface scrapings HM215439
Tooth surface scrapings HM215442
Tooth surface scrapings HM215448
Tooth surface scrapings HM215451
Conservation
100%
0%

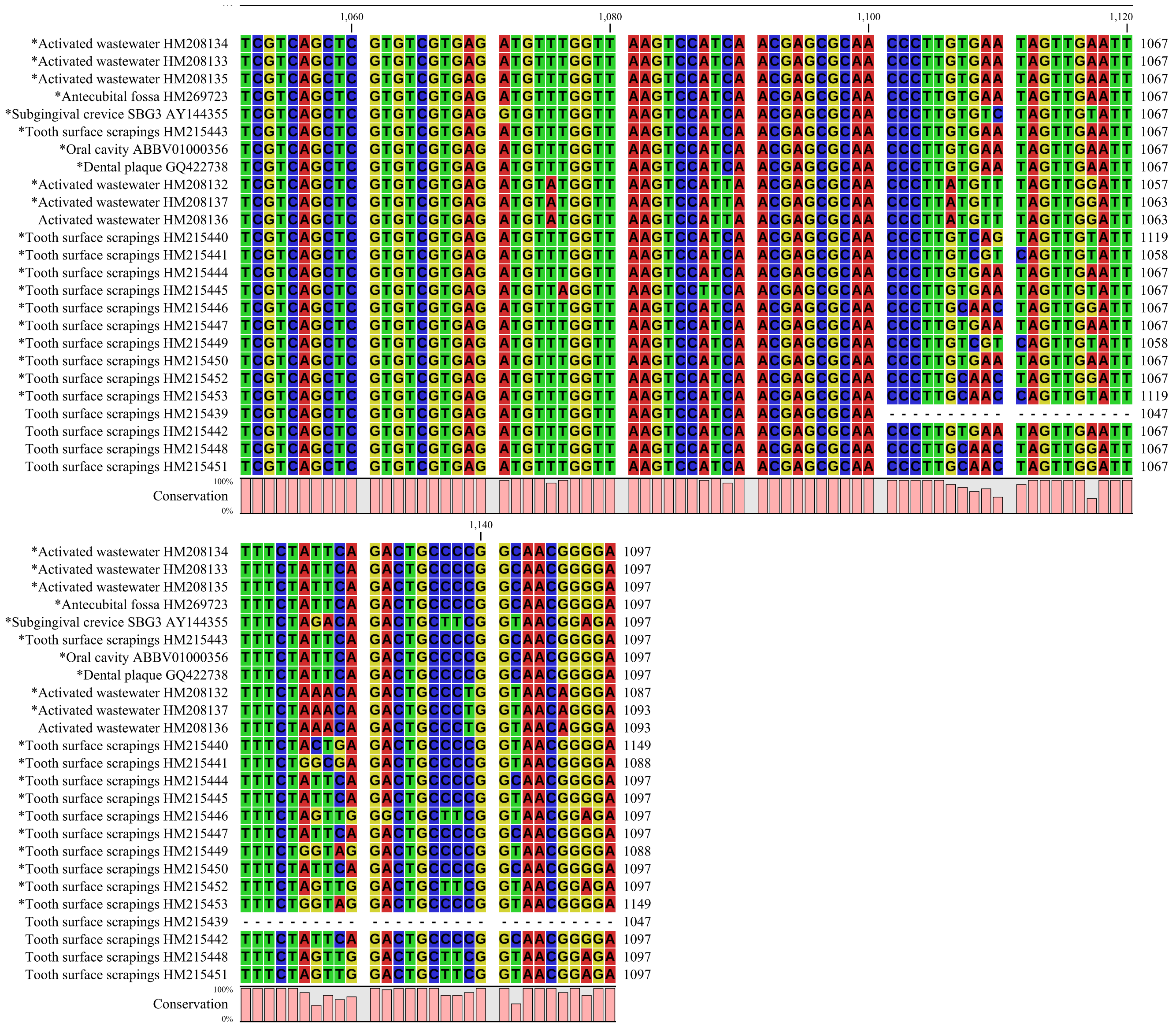
1,060
1,080
1,100
1,120
*Activated wastewater HM208134 TCGTCAGCTC GTGTCGTGAG ATGTTTGGTT AAGTCCATCA ACGAGCGCAA CCCTTGTGAA TAGTTGAATT 1067
*Activated wastewater HM208133 TCGTCAGCTC GTGTCGTGAG ATGTTTGGTT AAGTCCATCA ACGAGCGCAA CCCTTGTGAA TAGTTGAATT 1067
*Activated wastewater HM208135 TCGTCAGCTC GTGTCGTGAG ATGTTTGGTT AAGTCCATCA ACGAGCGCAA CCCTTGTGAA TAGTTGAATT 1067
*Antecubital fossa HM269723 TCGTCAGCTC GTGTCGTGAG ATGTTTGGTT AAGTCCATCA ACGAGCGCAA CCCTTGTGAA TAGTTGAATT 1067
*Subgingival crevice SBG3 AY144355 TCGTCAGCTC GTGTCGTGAG GTGTTTGGTT AAGTCCATCA ACGAGCGCAA CCCTTGTGTC TAGTTGTATT 1067
*Tooth surface scrapings HM215443 TCGTCAGCTC GTGTCGTGAG ATGTTTGGTT AAGTCCATCA ACGAGCGCAA CCCTTGTGAA TAGTTGAATT 1067
*Oral cavity ABBV01000356 TCGTCAGCTC GTGTCGTGAG ATGTTTGGTT AAGTCCATCA ACGAGCGCAA CCCTTGTGAA TAGTTGAATT 1067
*Dental plaque GQ422738 TCGTCAGCTC GTGTCGTGAG ATGTTTGGTT AAGTCCATCA ACGAGCGCAA CCCTTGTGAA TAGTTGAATT 1067
*Activated wastewater HM208132 TCGTCAGCTC GTGTCGTGAG ATGTATGGTT AAGTCCATTA ACGAGCGCAA CCCTTATGTT TAGTTGGATT 1057
*Activated wastewater HM208137 TCGTCAGCTC GTGTCGTGAG ATGTATGGTT AAGTCCATTA ACGAGCGCAA CCCTTATGTT TAGTTGGATT 1063
Activated wastewater HM208136 TCGTCAGCTC GTGTCGTGAG ATGTATGGTT AAGTCCATTA ACGAGCGCAA CCCTTATGTT TAGTTGGATT 1063
*Tooth surface scrapings HM215440 TCGTCAGCTC GTGTCGTGAG ATGTTTGGTT AAGTCCATCA ACGAGCGCAA CCCTTGTCAG TAGTTGTATT 1119
*Tooth surface scrapings HM215441 TCGTCAGCTC GTGTCGTGAG ATGTTTGGTT AAGTCCATCA ACGAGCGCAA CCCTTGTCGT CAGTTGTATT 1058
*Tooth surface scrapings HM215444 TCGTCAGCTC GTGTCGTGAG ATGTTTGGTT AAGTCCATCA ACGAGCGCAA CCCTTGTGAA TAGTTGAATT 1067
*Tooth surface scrapings HM215445 TCGTCAGCTC GTGTCGTGAG ATGTTAGGTT AAGTCCTTCA ACGAGCGCAA CCCTTGTGAA TAGTTGTATT 1067
*Tooth surface scrapings HM215446 TCGTCAGCTC GTGTCGTGAG ATGTTTGGTT AAGTCCATCA ACGAGCGCAA CCCTTGCAAC TAGTTGGATT 1067
*Tooth surface scrapings HM215447 TCGTCAGCTC GTGTCGTGAG ATGTTTGGTT AAGTCCATCA ACGAGCGCAA CCCTTGTGAA TAGTTGAATT 1067
*Tooth surface scrapings HM215449 TCGTCAGCTC GTGTCGTGAG ATGTTTGGTT AAGTCCATCA ACGAGCGCAA CCCTTGTCGT CAGTTGTATT 1058
*Tooth surface scrapings HM215450 TCGTCAGCTC GTGTCGTGAG ATGTTTGGTT AAGTCCATCA ACGAGCGCAA CCCTTGTGAA TAGTTGAATT 1067
*Tooth surface scrapings HM215452 TCGTCAGCTC GTGTCGTGAG ATGTTTGGTT AAGTCCATCA ACGAGCGCAA CCCTTGCAAC TAGTTGGATT 1067
*Tooth surface scrapings HM215453 TCGTCAGCTC GTGTCGTGAG ATGTTTGGTT AAGTCCATCA ACGAGCGCAA CCCTTGCAAC CAGTTGTATT 1119
Tooth surface scrapings HM215439 TCGTCAGCTC GTGTCGTGAG ATGTTTGGTT AAGTCCATCA ACGAGCGCAA ---------- ---------- 1047
Tooth surface scrapings HM215442 TCGTCAGCTC GTGTCGTGAG ATGTTTGGTT AAGTCCATCA ACGAGCGCAA CCCTTGTGAA TAGTTGAATT 1067
Tooth surface scrapings HM215448 TCGTCAGCTC GTGTCGTGAG ATGTTTGGTT AAGTCCATCA ACGAGCGCAA CCCTTGCAAC TAGTTGGATT 1067
Tooth surface scrapings HM215451 TCGTCAGCTC GTGTCGTGAG ATGTTTGGTT AAGTCCATCA ACGAGCGCAA CCCTTGCAAC TAGTTGGATT 1067
100%
Conservation
0%
1,140
*Activated wastewater HM208134 TTTCTATTCA GACTGCCCCG GCAACGGGGA 1097
*Activated wastewater HM208133 TTTCTATTCA GACTGCCCCG GCAACGGGGA 1097
*Activated wastewater HM208135 TTTCTATTCA GACTGCCCCG GCAACGGGGA 1097
*Antecubital fossa HM269723 TTTCTATTCA GACTGCCCCG GCAACGGGGA 1097
*Subgingival crevice SBG3 AY144355 TTTCTAGACA GACTGCTTCG GTAACGGAGA 1097
*Tooth surface scrapings HM215443 TTTCTATTCA GACTGCCCCG GCAACGGGGA 1097
*Oral cavity ABBV01000356 TTTCTATTCA GACTGCCCCG GCAACGGGGA 1097
*Dental plaque GQ422738 TTTCTATTCA GACTGCCCCG GCAACGGGGA 1097
*Activated wastewater HM208132 TTTCTAAACA GACTGCCCTG GTAACAGGGA 1087
*Activated wastewater HM208137 TTTCTAAACA GACTGCCCTG GTAACAGGGA 1093
Activated wastewater HM208136 TTTCTAAACA GACTGCCCTG GTAACAGGGA 1093
*Tooth surface scrapings HM215440 TTTCTACTGA GACTGCCCCG GTAACGGGGA 1149
*Tooth surface scrapings HM215441 TTTCTGGCGA GACTGCCCCG GTAACGGGGA 1088
*Tooth surface scrapings HM215444 TTTCTATTCA GACTGCCCCG GCAACGGGGA 1097
*Tooth surface scrapings HM215445 TTTCTATTCA GACTGCCCCG GTAACGGGGA 1097
*Tooth surface scrapings HM215446 TTTCTAGTTG GGCTGCTTCG GTAACGGAGA 1097
*Tooth surface scrapings HM215447 TTTCTATTCA GACTGCCCCG GCAACGGGGA 1097
*Tooth surface scrapings HM215449 TTTCTGGTAG GACTGCCCCG GTAACGGGGA 1088
*Tooth surface scrapings HM215450 TTTCTATTCA GACTGCCCCG GCAACGGGGA 1097
*Tooth surface scrapings HM215452 TTTCTAGTTG GACTGCTTCG GTAACGGAGA 1097
*Tooth surface scrapings HM215453 TTTCTGGTAG GACTGCCCCG GTAACGGGGA 1149
Tooth surface scrapings HM215439 ---------- ---------- ---------- 1047
Tooth surface scrapings HM215442 TTTCTATTCA GACTGCCCCG GCAACGGGGA 1097
Tooth surface scrapings HM215448 TTTCTAGTTG GACTGCTTCG GTAACGGAGA 1097
Tooth surface scrapings HM215451 TTTCTAGTTG GACTGCTTCG GTAACGGAGA 1097
100%
Conservation
0%
